# A systematic review and meta‐analysis of the relative benefit of dual versus single oral hypoglycaemic therapy following diagnosis with type 2 diabetes (T2D)

**DOI:** 10.1111/dme.70337

**Published:** 2026-05-21

**Authors:** Mairead Kelly, Sushant Saluja, Hugh Logan Ellis, Sarah Jamil, Martin B. Whyte, Adrian Heald

**Affiliations:** ^1^ Endocrinology & Diabetes Salford Royal NHS Foundation Trust Salford UK; ^2^ University of Manchester Manchester UK; ^3^ Kings College Hospital London UK; ^4^ University of Surrey Guildford UK

**Keywords:** dual therapy, HbA1c, monotherapy, SGLT2‐i, type 2 diabetes

## Abstract

**Introduction:**

Achieving and sustaining target glycated haemoglobin (HbA1c) levels is fundamental in the management of type 2 diabetes (T2D). We here aimed to assess whether initial dual oral therapy outperforms monotherapy in reaching glycaemic targets in patients with treatment‐naive or early‐stage T2D.

**Methods:**

This systematic review and meta‐analysis were registered with PROSPERO (CRD420251111096). Parallel‐group randomised controlled trials with a duration of at least 12 weeks were identified through searches of PubMed and the Cochrane Library spanning 2005 to 2025. Data at the trial arm level, including baseline and endpoint HbA1c values, were extracted for seven predefined comparisons and combined using a random‐effects inverse‐variance meta‐analysis in R version 4.3.1 (meta package). The primary outcome was the proportion of treatment arms achieving an HbA1c level of ≤7.5% (58 mmol/mol). Secondary outcomes included the proportions achieving HbA1c levels of ≤7.0% (53 mmol/mol) and ≤6.5% (48 mmol/mol), as well as mean differences in HbA1c levels.

**Results:**

A total of 20 trials, encompassing 37 treatment arms, were analysed. Dual combination therapy consistently demonstrated superior efficacy compared to monotherapy. The proportion of patients achieving HbA1c levels of ≤7.5% (58 mmol/mol) was 86% with initial dual therapy versus 82% with initial monotherapy (odds ratio [OR] 1.33, 95% confidence interval [CI] 1.20–1.47, *p* = 0.002). At the more stringent threshold of ≤7.0% (53 mmol/mol), the rates were 69% versus 64% (OR 1.27, *p* = 0.003), and at ≤6.5% (48 mmol/mol), 42% versus 39% (OR 1.18, *p* = 0.056). When comparing initial dual therapy to metformin monotherapy, the respective achievement rates were 86% versus 81% (OR 1.41, *p* = 0.001) for a target of ≤7.0% (53 mmol/mol).

The combination of metformin with a sodium–glucose cotransporter 2 inhibitor (SGLT2‐i) resulted in 88% reaching ≤7.5% (58 mmol/mol), compared to 81% with metformin alone (OR 1.55, *p* ≤ 0.001), a difference significant across all thresholds. Dual therapy containing SGLT‐2i achieved 87% at ≤7.5% (58 mmol/mol), compared with 82% with all monotherapies (OR 1.43, *p* ≤ 0.001). SGLT‐2i monotherapy itself led to 89% reaching the target, compared with 81% with metformin (OR 1.73, *p* ≤ 0.001). No significant difference in outcome was observed between dual and monotherapy involving SGLT2‐is (OR 0.87, *p* = 0.480).

The pooled final HbA1c values were 6.7% (50 mmol/mol) for dual therapy and 7.9% (63 mmol/mol) for monotherapy, corresponding to a mean difference of –0.45% (95% CI −0.60 to −0.25, *p* ≤ 0.001). Heterogeneity among studies was low to moderate (*I* ^2^ 25%–50%), and results remained consistent after excluding rosiglitazone arms.

**Conclusions:**

Initial dual therapy, particularly combining metformin with an SGLT2‐i, results in superior achievement of HbA1c targets across various thresholds compared to monotherapy. SGLT2‐i alone surpasses metformin alone in efficacy. Adding a second agent to SGLT2‐i did not provide additional glucose‐lowering benefit to SGLT2‐i monotherapy. Early initiation of SGLT2‐i monotherapy or combination therapy should be considered upon diagnosis of T2D.

**What's new?**

- This systematic review and meta‐analysis provide supportive evidence that initial dual oral therapy achieves better glycaemic control compared to monotherapy across several HbA1c thresholds in treatment‐naive and early T2D patients.
- The combination of metformin and SGLT2‐i demonstrated the greatest efficacy (≤7.5%: OR 1.55, *p* ≤ 0.001), while SGLT2‐i monotherapy exceeded metformin alone (OR 1.73, *p* ≤ 0.001).
- SGLT2‐i dual therapy (i.e. SGLT2‐i plus another glucose‐lowering agent) showed no added advantage over SGLT2‐i inhibitor monotherapy, establishing SGLT2‐is as a potent initial agent. Thus, these findings may have important clinical implications.

## 1 INTRODUCTION

Achieving and sustaining target glycated haemoglobin (HbA1c) levels is fundamental in the management of type 2 diabetes (T2D).^1^ It represents the key modifiable factor influencing microvascular, macrovascular and renal complications while supporting quality of life and reducing long‐term disability. For many years, metformin has been the first‐line pharmacotherapy preferred in the United Kingdom, Europe and numerous other regions globally—unless contraindicated due to renal impairment (eGFR ≤ 30 mL/min/1.73 m^2^) or intolerable gastrointestinal side effects—paired with comprehensive lifestyle interventions including dietetic advice, regular physical activity and behavioural modifications.^1^, ^2^

The multifaceted treatment requirements for T2D extend well beyond glycaemic control, encompassing intensive management of the interconnected cardiovascular risk factors: dyslipidaemia, hypertension and central obesity.^1^ However, monotherapy with metformin consistently fails to achieve target HbA1c levels (≤7.0% or 53 mmol/mol for most patients; ≤6.5% or 48 mmol/mol for select younger individuals without comorbidities) in 40%–60% of cases, with success rates dropping below 30 percent among those with baseline HbA1c values of 8.0% or higher (≥64 mmol/mol).^3^ Failure to achieve rapid glycaemic control in T2D may be compounded by therapeutic inertia, which delays timely intensification of therapy, allowing hyperglycaemia to persist.^4^ Prolonged exposure to chronic hyperglycaemia worsens β‐cell dysfunction and insulin resistance, making subsequent glucose control increasingly difficult and less responsive to treatment.^5^

Contemporary approaches to therapy are experiencing significant change. The 2025 American Diabetes Association Standards of Care now recognise sodium‐glucose co‐transporter‐2 (SGLT2) inhibitors as valid first‐line options, rather than relying solely on metformin monotherapy, for patients with established diabetic kidney disease or atherosclerotic cardiovascular disease.^6^ This shift is based on their ability to reduce rates of heart failure hospitalisations (HR 0.67–0.79), delay progression to end‐stage kidney disease (HR 0.61–0.71), and lower cardiovascular mortality, as well as to lower HbA1c by 0.5%–0.9%.^7^, ^8^ Increasing evidence from randomised controlled trials questions the traditional stepwise escalation model, suggesting that initial dual oral therapy may provide a greater, faster and more sustained glycaemic response, thus avoiding therapeutic inertia.

This PROSPERO‐registered systematic review and meta‐analysis (CRD420251111096) synthesises current evidence from 20 parallel‐group randomised controlled trials, involving 37 independent treatment arms, to assess whether initiating dual oral pharmacotherapy at the time of T2D diagnosis results in better HbA1c target achievement, at thresholds of ≤7.5%, ≤7.0%, and ≤6.5%, compared to monotherapy approaches.

By stratifying analyses across different monotherapy classes, metformin‐based combinations and sodium–glucose cotransporter 2 inhibitor (SGLT2‐i)–inclusive regimens, covering baseline HbA1c levels from 6.5% (48 mmol/mol) to 11.0% (97 mmol/mol) and trial durations of 12 to 52 weeks, this study allowed us to consider a range of treatment regimens to address the question of whether dual treatment is better than monotherapy?

## 2 METHODS

### 2.1 Protocol and reporting

**FIGURE 1:**
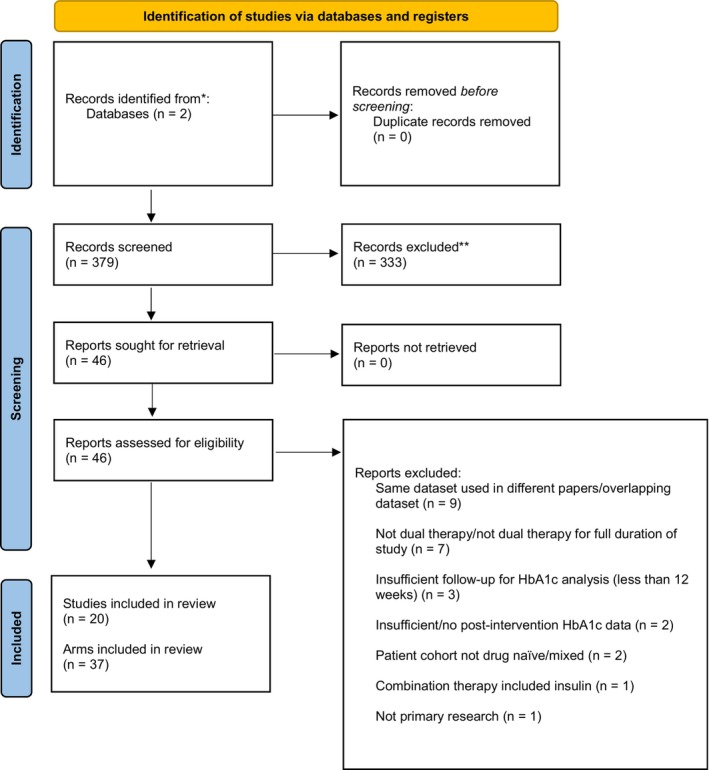
PRISMA 2020 flow diagram of study selection for the systematic review and meta‐analysis evaluating initial dual versus single oral hypoglycaemic therapy in treatment‐naive or early type 2 diabetes. The diagram illustrates the identification of 379 records through database searches of PubMed and Cochrane Library (2005–2025), screening of titles and abstracts by three independent reviewers, full‐text assessment of 46 articles for eligibility and final inclusion of 20 parallel‐group randomised controlled trials encompassing 37 independent treatment arms, after exclusion based on prespecified criteria (treatment duration ≤12 weeks, insulin use, rosiglitazone combinations, absence of HbA1c endpoint data, or other factors detailed in the Methods section). This rigorous selection process provides the evidential foundation for the pooled proportion meta‐analyses of HbA1c target attainment (≤6.5%, ≤7.0%, ≤7.5%) across monotherapy and dual‐therapy strategies, as summarised in Table 1 and detailed in the Results.^9^ This work is licensed under CC BY 4.0.

This systematic review and meta‐analysis were prospectively registered in PROSPERO (CRD420251111096) and conducted in accordance with PRISMA guidelines using a predefined protocol. The PRISMA flow diagram (Figure 1) illustrates the process of identifying, screening, assessing eligibility and including studies.^9^

### 2.2 Data sources and search strategy

Electronic searches of PubMed and the Cochrane Library were performed for the dates 1 January 2005 to 1 December 2025, without language restrictions. Search terms combined subject headings and free‐text words related to type 2 diabetes mellitus (T2D), newly diagnosed or pharmacotherapy‐naïve status, oral hypoglycaemic agents (including metformin, dipeptidyl‐peptidase IV (DPP‐4) inhibitors, SGLT‐2is, sulfonylureas, thiazolidinediones, meglitinides, alpha‐glucosidase inhibitors, bile acid sequestrants and traditional Chinese medicines) and randomised controlled trials. References from eligible trials and relevant systematic reviews were also screened for additional studies.

A targeted search of PubMed (2005–2025) was additionally conducted to identify randomised controlled trials specifically comparing initial dual oral therapy with a DPP4‐I plus metformin against monotherapy (metformin, DPP‐4 inhibitor, or sulfonylurea) or less intensive combinations in treatment‐naive or early‐stage T2D; these trials constituted the core dataset for primary analyses.

### 2.3 Study selection and eligibility criteria

Three reviewers (MK, SJ, HLE) independently screened titles and abstracts of all retrieved records, followed by full‐text assessments of potentially relevant articles. Disagreements were resolved through discussion or, if necessary, consultation with a fourth reviewer (AH). Eligible studies were randomised, open‐label or blinded, parallel‐group trials involving adults with T2D who were newly diagnosed, pharmacotherapy‐naïve, or had an adequate washout period from previous glucose‐lowering therapy. Trials needed to assess initial combination oral hypoglycaemic therapy versus monotherapy or stepwise escalation, include at least 12 weeks of follow‐up, and report baseline and post‐intervention glycated haemoglobin (HbA1c) levels, or HbA1c change.

Combination therapy could involve any two oral agents from predefined classes (biguanides, dipeptidyl peptidase‐4 inhibitors (DPP‐4is), SGLT2‐is, thiazolidinediones, sulfonylureas, meglitinides, alpha‐glucosidase inhibitors, bile acid sequestrants or traditional Chinese medicines). Studies were excluded if they were extension trials, had treatment durations shorter than 12 weeks, failed to report HbA1c changes or involved insulin initiation as part of the regimen. Studies including rosiglitazone in combination therapy were reviewed but were not included in the final analysis. The PRISMA flow diagram indicates that 379 records were screened, 46 full‐text articles assessed for eligibility and 20 trials (37 arms) were included (Figure 1).

### 2.4 Data extraction and outcomes

Three reviewers (MK, SJ, HLE) independently extracted data using a standardised data‐collection form, with a third reviewer (AH) verifying entries against original publications. Extracted variables included first author, year, country, study design, baseline participant characteristics (mean age, sex distribution, baseline HbA1c), sample size per arm, details of interventions and comparators (drug class, dose, schedule), study duration and primary outcomes. For metformin, we recorded the reported dose and titration schedule when available; however, because dosing categories were not consistently reported across all trials, metformin monotherapy arms were analysed as a single comparator group rather than stratified by dose.

For each trial arm, baseline and endpoint mean HbA1c and standard deviations (when available) were recorded, along with the absolute change in HbA1c. When responder proportions at specific HbA1c thresholds were reported only as percentages, absolute numbers were reconstructed from the reported percentages using the randomised denominator, adopting an intention‐to‐treat approach. The primary endpoint was the proportion of study arms achieving a final mean HbA1c of ≤7.5% (58 mmol/mol) after ≥12 weeks of therapy. Secondary endpoints included the proportions reaching ≤7.0% (53 mmol/mol) and ≤6.5% (48 mmol/mol), as well as the pooled mean difference in final HbA1c between treatment strategies.

### 2.5 Risk of bias assessment

Risk of bias for each trial was evaluated using the Cochrane risk‐of‐bias tool version 2 (RoB 2), assessing random sequence generation, allocation concealment, blinding of participants and personnel, blinding of outcome assessment, incomplete outcome data, selective reporting and other bias sources.^10^ An overall judgment (low risk, some concerns, high risk) was provided for each study; results are summarised in Figure S1.

### 2.6 Statistical analysis

All analyses were conducted using R version 4.3.1 with the *meta* package. A dataset of study arms was compiled from included trials and grouped into seven prespecified comparisons: (1) all dual therapy versus all monotherapies; (2) dual therapy versus metformin monotherapy; (3) dual therapy with metformin plus other oral agents versus metformin alone; (4) SGLT2‐i plus metformin versus metformin alone; (5) SGLT2‐i containing dual therapy versus all monotherapy; (6) SGLT2‐i dual therapy versus SGLT2‐i monotherapy; and (7) SGLT2‐i monotherapy versus metformin monotherapy. For analysis, individual SGLT2‐is were grouped into a single drug class because the number of arms for any one agent was insufficient for reliable agent‐specific meta‐analyses across all HbA1c thresholds and current clinical guidelines generally position SGLT2‐i as a class‐level treatment option.

Meta‐analyses employed inverse‐variance random‐effects models to account for between‐study heterogeneity. For binary outcomes (proportion achieving target HbA1c at each threshold), pooled odds ratios (ORs) with 95% confidence intervals (CIs) and *p*‐values were calculated. Continuous outcomes (final HbA1c) were summarised as pooled mean differences. Heterogeneity was quantified using *I* ^2^ and *τ* ^2^ statistics. Sensitivity analyses excluding rosiglitazone‐containing arms were performed to assess robustness, with any notable changes documented qualitatively.

The unit of analysis for all meta‐analyses was the individual trial arm. We constructed a dataset of study arms and mapped each arm to a single prespecified comparison (e.g., metformin plus SGLT2‐i vs. metformin monotherapy, SGLT2‐i monotherapy vs. metformin monotherapy or SGLT2‐i‐based dual therapy vs. all monotherapies). Within any given meta‐analytic contrast, each arm was entered once, and no comparator arm was duplicated or split across multiple contrasts, thereby avoiding double‐counting of shared control groups. Because arms from multi‐arm trials were assigned uniquely within each comparison, additional statistical adjustment for within‐study correlation of shared comparators was not required. All models used inverse‐variance random‐effects pooling to account for residual between‐study heterogeneity.

## 3 RESULTS

Across the 20 randomised controlled trials included in the meta‐analysis (Table 1), comprising 37 treatment arms and 11,478 participants, aggregate baseline characteristics are summarised in Table 2. These studies enrolled adults with newly diagnosed or early T2D, with a mean baseline HbA1c of 8.5% (SD 1.0%) and follow‐up durations of 12 to 52 weeks. These characteristics provide the clinical context for the pooled analyses of HbA1c target attainment at 6.5%, 7.0% and 7.5% across different initial pharmacotherapy strategies (Table 3).

**TABLE 1:** A summary of included studies, design, interventions, comparators and primary outcomes.

Author	Year of study	Title of study	Baseline population	% male	Country of study	Study design	Interventions	Comparator	Intervention/comparator	Study duration	Primary measures
Amblee et al.^21^	2015	A combination of saxagliptin and metformin is effective as initial therapy in new‐onset type 2 diabetes mellitus with severe hyperglycemia	Newly diagnosed T2DM	81	USA	Open‐label RCT	Saxagliptin 5 mg + metformin 200 mg daily	Glipizide XL 10 mg daily	50/50	12 weeks	Fasting/premeal glucose levels, return acute‐care site visits
Chen et al.^22^	2010	Effects of metformin plus gliclazide compared with metformin alone on circulating endothelial progenitor cells in type 2 diabetic patients	Newly diagnosed T2DM	53.2	China	Open‐label RCT	Gliclazide (modified release, 30–60 mg/day) plus metformin (250–1000 mg/day)	Metformin 2500 mg daily	23/24	16 weeks	Circulating endothelial progenitor cells, Progression of diabetic vascular complications
Chen et al.^23^	2022	Saxagliptin combined with additional oral antihyperglycaemic agents in drug‐naive diabetic patients with high glycosylated haemoglobin: A 24‐week, multicentre, randomized, open‐label, active parallel‐controlled group clinical trial in China (SUCCESS)	Newly diagnosed T2DM	69.8	China	Open‐label, active parallel‐controlled multicentre RCT	Saxagliptin 5 mg + metformin 2000 mg, Saxagliptin 5 mg + acarbose 300 mg, saxagliptin 5 mg + gliclazide 120 mg	None	648/0	24 weeks	Absolute change in HbA1c from baseline
Chou et al.^24^	2007	Initial treatment with fixed‐dose combination rosiglitazone/glimepiride in patients with previously untreated type 2 diabetes	T2DM patients who were managed by dietary changes alone, or had not had any oral hypoglycaemic agents or insulin for over 15 days in the preceding 4 month period	58.6	Multinational	Randomised, double‐blind, parallel‐group study	Rosiglitazone/glimepiride (up to 4 mg/4 mg daily), rosiglitazone/glimepiride (up to 8 mg/8 mg daily)	Glimepiride 4 mg daily, rosiglitazone 8 mg od	388/393	28 weeks	Absolute change in HbA1c from baseline
Dou et al.^25^	2018	Efficacy and safety of saxagliptin in combination with metformin as initial therapy in Chinese patients with type 2 diabetes: Results from the START study, a multicentre, randomized, double‐blind, active‐controlled, phase 3 trial	Pharmacotherapy‐naive T2DM patients	66.5	China	Randomised, double‐blind, active‐controlled phase 3 trial	Saxagliptin 5 mg + mMetformin	Metformin + placebo, saxagliptin + placebo	210/420	24 weeks	Changes in mean fasting plasma glucose, 120‐min post‐prandial glucose and 180‐min post‐prandial glucose area under the curve, therapeutic glycaemic response
Haak et al.^26^	2011	Initial combination of linagliptin and metformin improves glycaemic control in type 2 diabetes: a randomized, double‐blind, placebo‐controlled study	T2DM patients with HbA1c ≤11, and a washout period of 4 weeks from oral hypoglycaemic agents	52.7	Multinational	Multi‐centre, double‐blind, randomized, placebo‐controlled, parallel‐group phase 3 clinical trial	Linagliptin 2.5 + metformin 1000 mg, linagliptin 2.5 + metformin 2000 mg	Linaglipin 5 mg, placebo, metformin 100 mg, metformin 2000 mg	286/505	24 weeks	Mean endpoint HbA1c from baseline
Hadjadj et al.^27^	2014	initial combination of empagliflozin and metformin in patients with type 2 diabetes	Pharmacotherapy‐naive T2DM patients	56.3	Multinational	Multi‐centre, double‐blind, randomized, placebo‐controlled, parallel‐group phase 3 clinical trial	Empagliflozin 12.5 mg b.i.d. + metformin 1000 mg b.i.d, empagliflozin 12.5 mg b.i.d. + metformin 500 mg b.i.d, empagliflozin 5 mg b.i.d. + metformin 1000 mg b.i.d, empagliflozin 5 mg b.i.d. + metformin 500 mg b.i.d	Metformin 1000 mg b.i.d. monotherapy, metformin 500 mg b.i.d. monotherapy, empagliflozin 25 mg q.d. monotherapy, empagliflozin 10 mg q.d. monotherapy	662/665	24 weeks	Absolute change in HbA1c from baseline
Jadzinsky et al.^28^	2009	Saxagliptin given in combination with metformin as initial therapy improves glycaemic control in patients with type 2 diabetes compared with either monotherapy: a randomized controlled trial	Pharmacotherapy‐naive T2DM patients	49.2	Multinational	Multi‐centre, double‐blind, randomized, active‐controlled phase 3 clinical trial	Saxagliptin 5 mg + metformin IR 500 mg, saxagliptin 10 mg + metformin IR 500 mg	metformin IR 500 mg + placebo, saxagliptin 10 mg + placebo	643/663	24 weeks	Absolute change in HbA1c from baseline
Ji et al.^29^	2016	Randomized clinical trial of the safety and efficacy of sitagliptin and metformin co‐administered to Chinese patients with type 2 diabetes mellitus	T2DM patients who were managed by dietary changes alone, or had not had any oral hypoglycaemic agents or insulin for within an 8 week washout period	61.4	China	Multi‐centre, double‐blind, randomised, placebo‐controlled, parallel group clinical trial	Sitagliptin 50 mg plus metformin 500 mg, sitagliptin 50 mg plus metformin 850 mg	Metformin 1000 mg, metformin 1700 mg, placebo, sitagliptin 100 mg	247/497	24 weeks	Absolute change in HbA1c from baseline
Ji et al.^30^	2017	Efficacy and safety of fixed dose combination therapy, alogliptin plus metformin, in Asian patients with type 2 diabetes: A phase 3 trial	Pharmacotherapy‐naïve T2DM patients	56.6	China	Multi‐centre, double‐blind, randomized, placebo‐controlled phase 3 clinical trial	Alogliptin 25 mg + metformin 1000 mg	Metformin 1000 mg, alogliptin 25 mg, placebo	158/485	26 weeks	Absolute change in HbA1c from baseline
Ji et al.^31^	2015	Efficacy and safety of linagliptin co‐administered with low‐dose metformin once daily versus high‐dose metformin twice daily in treatment‐naive patients with type 2 diabetes: a double‐blind randomized trial	T2DM patients who were managed by dietary changes alone, or had not had any oral hypoglycaemic agents or insulin for a 12‐week washout period	47.4	Multinational	Multi‐centre, double‐blind, randomized, parallel‐group, active‐controlled phase 4 clinical trial	Linagliptin 5 mg + metformin 1000 mg	Metformin 2000 mg	344/345	14 weeks	Absolute change in HbA1c from baseline
Jianfang et al.^32^	2023	Effect and safety of pioglitazone‐metformin tablets in the treatment of newly diagnosed type 2 diabetes patients with nonalcoholic fatty liver disease in Shaanxi Province: A randomized, double‐blinded, double‐simulated multi‐center study	Newly diagnosed T2DM with fatty liver disease	71.6	China	Multi‐centre, double‐blind, randomised, double‐simulation, positive‐drug control clinical trial	Pioglitazone 15 mg + metformin 1000 mg	Metformin 1000 mg	60/60	24 weeks	Liver fat content, γ‐GT level
Lewin et al.^33^	2015	Initial combination of empagliflozin and linagliptin in subjects with type 2 diabetes	T2DM patients who were managed by dietary changes alone, or had not had any oral hypoglycaemic agents or insulin for a 12 week washout period (metformin arms not used in this study)	53.8	Multinational	Multi‐centre, double‐blind, randomised, parallel‐group phase 3 clinical trial	Empagliflozin 25 mg/linagliptin 5 mg, empagliflozin 10 mg/linagliptin 5 mg	Linagliptin 5 mg, empagliflozin 25 mg, empagliflozin 10 mg	269/398	52 weeks	Absolute change in HbA1c from baseline
Mu et al.^34^	2017	Efficacy and safety of linagliptin/metformin single‐pill combination as initial therapy in drug‐naive Asian patients with type 2 diabetes	Pharmacotherapy‐naïve T2DM patients	59.9	China	Multi‐centre, double‐blind, randomised, double‐dummy, parallel group, phase 3 trial	Linagliptin 2.5/metformin 500 mg bd, linagliptin 2.5/metformin 1000 mg bd	Metformin 500 mg bd, metformin 1000 mg bd, linagliptin 5 mg	292/336	24 weeks	Absolute change in HbA1c from baseline
Pan et al.^35^	2021	The effectiveness of traditional Chinese medicine Jinlida granules on glycemic variability in newly diagnosed type 2 diabetes: a double‐blinded, randomized trial	Newly diagnosed T2DM	68.1	China	Double‐blind, randomised, placebo‐controlled, parallel‐group clinical trial	Jinlinda granules (1 sachet) + metformin 1500 mg	Metformin 1500 mg + placebo, placebo, Jinlinda	33/105	16 weeks	Changes in HbA1c, traditional Chinese medicine symptom score, and CGM parameters
Perez et al.^36^	2009	Efficacy and safety of pioglitazone/metformin fixed‐dose combination therapy compared with pioglitazone and metformin monotherapy in treating patients with T2DM	T2DM patients who were managed by dietary changes alone, or had not had any oral hypoglycaemic agents or insulin for a 12‐week washout period	42.3	USA	Multi‐centre, double‐blind, randomised, parallel‐group controlled trial	Pioglitazone 15 mg + metformin 850 mg bd	Pioglitazone 15 mg bd, metformin 850 mg bd	210/399	24 weeks	Absolute change in HbA1c from baseline
Rosenstock et al.^37^	2016	Initial combination therapy with canagliflozin plus metformin versus each component as monotherapy for drug‐naive type 2 diabetes	Pharmacotherapy‐naive T2DM patients	48	Multinational	Multi‐centre, double‐blind, randomised, parallel‐group phase 3 clinical trial	Canagliflozin 100 mg + metformin XR, canagliflozin 300 mg + metformin XR	Metformin XR, canagliflozin 100 mg, canagliflozin 300 mg	464/712	26 weeks	Absolute change in HbA1c from baseline
Rosenstock et al.^38^	2010	Initial combination therapy with metformin and colesevelam for the achievement of glycemic and lipid goals in early type 2 diabetes	T2DM patients who were managed by dietary changes alone, or had not had any oral hypoglycaemic agents or insulin for a 12‐week washout period	38.2	Multinational	Multi‐centre, double‐blind, randomised, parallel‐group clinical trial	Colesevalam 3.75 g daily + metformin 1700 mg	Metformin 1700 mg + placebo	145/141	16 weeks	Absolute change in HbA1c from baseline
Rosenstock et al.^39^	2010	Initial combination therapy with alogliptin and pioglitazone in drug‐naive patients with type 2 diabetes	T2DM patients who were managed by dietary changes alone, or had not had any oral hypoglycaemic agents or insulin for a 12‐week washout period	48.9	Multinational	Multi‐centre, double‐blind, randomised, parallel‐group phase 3 clinical trial	Alogliptin 12.5 mg daily + pioglitazone 30 mg daily, alogliptin 25 mg daily + pioglitazone 30 mg daily	Alogliptin 25 mg, pioglitazone 30 mg	327/327	26 weeks	Absolute change in HbA1c from baseline
Ross et al.^40^	2015	Initial combination of linagliptin and metformin compared with linagliptin monotherapy in patients with newly diagnosed type 2 diabetes and marked hyperglycaemia: a randomized, double‐blind, active‐controlled, parallel group, multinational clinical trial	T2DM patients who were managed by dietary changes alone, or had not had any oral hypoglycaemic agents or insulin for a 12‐week washout period	46.2	Multinational	Multi‐centre, double‐blind, randomised, active‐controlled, parallel‐group clinical trial	Linagliptin 5 mg + metformin 1500‐2000 mg daily	Linagliptin 5 mg	159/157	24 weeks	Absolute change in HbA1c from baseline
Tao et al.^41^	2018	Comparison of glycemic control and beta‐cell function in new onset T2DM patients with PCOS of metformin and saxagliptin monotherapy or combination treatment	Newly diagnosed T2DM with PCOS	0	China	Randomised, parallel, open‐label study	Metformin 2000 mg/day + saxagliptin 5 mg/day	Metformin 2000 mg, saxagliptin 5 mg/day	21/42	24 weeks	Change in glycaemic control and beta‐cell function
Wang et al.^42^	2011	Randomized study of repaglinide alone and in combination with metformin in Chinese subjects with type 2 diabetes naive to oral antitherapy	Pharmacotherapy‐naive T2DM patients	72.5	China	Randomised, parallel, open‐label study	Repaglinide 1 mg + Metformin 500 mg tds	Repaglinide 1 mg tds	218/214	16 weeks	Absolute change in HbA1c from baseline
Yoo et al.^43^	2021	Long‐term glycaemic durability of early combination therapy strategy versus metformin monotherapy in Korean patients with newly diagnosed type 2 diabetes mellitus	Newly diagnosed T2DM	51.3	Korea	Double‐blind, randomised, parallel‐group, phase 4 trial	Vidagliptin 50 mg bd + metfomin 2000 mg daily	Metformin 2000 mg daily + placebo	22/17	26 weeks	Time to treatment failure

**TABLE 2:** Summary data from per‐arm extraction across 21 randomised controlled trials.

Characteristic	Dual Therapy Arms (*n* = 23 arms, *N* = 5,958)	Monotherapy Arms (*n* = 14 arms, *N* = 5,520)	All Arms (*n* = 37 arms, *N* = 11,478)
Total participants (*N*)	5,958	5,520	11,478
Mean baseline HbA1c (%)	8.6 (range 6.5–10.9)	8.4 (range 6.5–11.1)	8.5 (range 6.5–11.1)
Mean age (years)	55.3 (SD 5.2)	55.8 (SD 5.4)	55.5 (SD 5.3)
% Male	58.2%	56.7%	57.5%
Mean T2D duration	Newly diagnosed/drug‐naïve	Newly diagnosed/drug‐naïve	Newly diagnosed/drug‐naïve
Mean follow‐up (weeks)	24.2 (range 12–52)	23.8 (range 12–52)	24.0 (range 12–52)

*Note*: Means are weighted by sample size per arm. Age and HbA1c SDs calculated from reported study values.

**TABLE 3:** Outcomes of the meta‐analysis of initial oral therapy for type 2 diabetes.

Comparison (Group 1 vs Group 2)	HbA1c target	Group 1: % at target	Group 2: % at target	*p*‐value	OR (Group 1 vs Group 2)
All dual therapy vs all monotherapy	≤6.5%	42.3	38.8	0.056	1.18
	≤7.0%	69.2	63.9	0.003	1.27
	≤7.5%	86.0	81.7	0.002	1.33
All dual therapy vs metformin monotherapy	≤6.5%	42.3	37.5	0.063	1.23
	≤7.0%	69.2	62.8	0.001	1.33
	≤7.5%	86.0	80.8	0.001	1.41
Dual metformin + any other agent vs metformin monotherapy	≤6.5%	42.0	37.5	0.079	1.20
	≤7.0%	69.0	62.8	0.001	1.31
	≤7.5%	85.8	80.8	0.002	1.39
Dual metformin + SGLT2 inhibitor vs metformin monotherapy	≤6.5%	43.7	37.5	0.030	1.29
	≤7.0%	71.3	62.8	≤0.001	1.47
	≤7.5%	87.6	80.8	≤0.001	1.55
Dual SGLT2 inhibitor + any agent vs all monotherapy	≤6.5%	42.9	38.8	0.173	1.19
	≤7.0%	70.4	63.9	≤0.001	1.35
	≤7.5%	86.9	81.7	≤0.001	1.43
Dual SGLT2 inhibitor + any agent vs SGLT2 monotherapy	≤6.5%	42.9	44.9	0.640	0.92
	≤7.0%	70.4	72.7	0.455	0.89
	≤7.5%	86.9	88.5	0.480	0.87
SGLT2 monotherapy vs metformin monotherapy	≤6.5%	44.9	37.5	0.028	1.36
	≤7.0%	72.7	62.8	≤0.001	1.57
	≤7.5%	88.5	80.8	≤0.001	1.73

*Note*: The table summarises, for each study arm, sample size, mean age, sex distribution, baseline HbA1c, trial duration, and the specific pharmacological regimen (monotherapy versus dual therapy, including metformin‐based and SGLT‐2 is containing combinations), thereby providing the clinical and methodological context for the pooled analyses of HbA1c target achievement at ≤6.5%, ≤7.0%, and ≤7.5%.

For all monotherapy compared with all dual‐therapy arms, the pooled estimated proportion of patients achieving HbA1c ≤ 6.5% (48 mmol/mol) was 38.8% with monotherapy versus 42.3% with dual therapy (*p* = 0.056), representing a numerically favourable but non‐significant difference in favour of dual therapy (Table 3). At HbA1c ≤ 7.0% (53 mmol/mol), 63.9% of monotherapy arms versus 69.2% of dual‐therapy arms reached target (*p* = 0.003), and at ≤7.5% (58 mmol/mol) the corresponding figures were 81.7% versus 86.0% (*p* = 0.002).

When all dual‐therapy regimens were explicitly compared with metformin monotherapy, metformin alone achieved an estimated 37.5% of patients at HbA1c ≤ 6.5% (48 mmol/mol) versus 42.3% for dual therapy (*p* = 0.063). At HbA1c ≤ 7.0% (53 mmol/mol), 62.8% versus 69.2% achieved target (*p* = 0.001), and at ≤7.5% (58 mmol/mol), 80.8% versus 86.0% reached target (*p* = 0.001) (Table 3).

Restricting the analysis to dual‐therapy regimens comprising metformin plus any other oral agent yielded closely similar patterns. For ‘metformin plus any agent vs metformin alone’, the estimated proportion achieving HbA1c ≤ 6.5% (48 mmol/mol) was 42.0% for metformin‐based dual therapy compared with 37.5% for metformin monotherapy (*p* = 0.079). At HbA1c ≤ 7.0% (53 mmol/mol), 69.0% versus 62.8% achieved target (*p* = 0.001), and at ≤7.5% (58 mmol/mol), 85.8% versus 80.8% (*p* = 0.002).

In analyses focusing specifically on regimens combining metformin with an SGLT2‐i, dual therapy with metformin plus SGLT2‐i was consistently superior to metformin monotherapy across all three thresholds (Table 3). At HbA1c ≤ 6.5% (48 mmol/mol), the estimated proportions achieving target were 37.5% with metformin alone versus 43.7% with metformin plus SGLT2‐i (*p* = 0.030). At HbA1c ≤ 7.0% (53 mmol/mol), 62.8% versus 71.3% reached target (*p* ≤ 0.001) and at ≤7.5% (58 mmol/mol), 80.8% versus 87.6% (*p* ≤ 0.001).

Broader comparisons of SGLT2‐i ‐containing dual therapy versus all monotherapy arms showed a similar pattern. For ‘SGLT2‐based dual therapy vs all monotherapy’, estimated attainment of HbA1c ≤ 6.5% (48 mmol/mol) was 38.8% with monotherapy versus 42.9% with SGLT2‐i ‐based dual therapy (*p* = 0.173). At HbA1c ≤ 7.0% (53 mmol/mol), 63.9% versus 70.4% achieved target (*p* ≤ 0.001) and at ≤7.5% (58 mmol/mol), 81.7% versus 86.9% (*p* ≤ 0.001).

By contrast, when dual regimens (comprising SGLT2‐i plus another agent) were compared directly with SGLT2‐i monotherapy, there was no evidence of additional glycaemic benefit from adding a second agent. For ‘SGLT2 as part of dual therapy vs SGLT2 monotherapy’, the estimated proportion achieving HbA1c ≤ 6.5% (48 mmol/mol) was 42.9% with dual therapy versus 44.9% with SGLT2‐i monotherapy (*p* = 0.640); for HbA1c ≤ 7.0% (53 mmol/mol), 70.4% versus 72.7% achieved target (*p* = 0.455); and for ≤7.5% (58 mmol/mol), 86.9% versus 88.5% (*p* = 0.480).

Finally, a direct comparison of SGLT2‐i monotherapy with metformin monotherapy demonstrated a consistent, statistically significant advantage of SGLT2 inhibitors across all targets. For ‘metformin monotherapy vs SGLT2‐i monotherapy’, at HbA1c ≤ 6.5% (48 mmol/mol), 37.5% versus 44.9% of patients achieved the threshold (metformin vs. SGLT2‐i; *p* = 0.028). At HbA1c ≤ 7.0% (53 mmol/mol), 62.8% versus 72.7% reached target (*p* ≤ 0.001), and at ≤7.5% (58 mmol/mol), 80.8% versus 88.5% (*p* ≤ 0.001).

All these comparisons were repeated after excluding the two rosiglitazone‐containing arms listed in Table 4, to reflect contemporary prescribing practice. The pooled estimated proportions and *p*‐values changed only minimally and no comparison altered its qualitative interpretation: comparisons that were previously statistically significant (for example, dual vs. metformin alone at 7.0% (53 mmol/mol) and 7.5% (58 mmol/mol); metformin plus SGLT2‐i versus metformin alone at all three thresholds; SGLT2‐i monotherapy versus metformin monotherapy at all three thresholds) remained significant. Those that were previously non‐significant (such as SGLT2‐i‐based dual vs. SGLT2‐i monotherapy, or differences at the 6.5% (48 mmol/mol) threshold in the broader dual‐vs‐monotherapy contrasts) remained non‐significant. Exclusion of rosiglitazone, therefore, did not materially affect the conclusions of the analyses presented in the Results section.

**TABLE 4:** Summary of intervention arms – excluding trials that included rosiglitazone, with standard deviation where provided.

Author	Year of study	Study arm	HbA1c pre‐intervention (SD)	HbA1c post‐intervention (SD)	HbA1c change (SD)
Amblee et al^21^	2016	Saxagliptin 5mg + metformin up to 2000mg	10.9 (1.4)	6.8	4.1
		Glipizide 10mg	11.1 (1.39)	6.9	4.2
Anholm et al	2019	Liraglutide 1.8mg od + metformin 2000mg	6.5 (0.5)	6.1 (0.5)	0.4
		Placebo + metformin 2000mg	6.5 (0.5)	6.4 (0.4)	0.1
Chen LL, et al^22^	2010	Gliclazide (modified release, 30–60 mg/day) plus metformin (250–1000 mg/day)	7.6 (1)	6 (0.6)	1.6
		Metformin 500–2500 mg/day	7.6 (1)	6.1 (0.5)	1.5
Chen, X et al^23^	2023	Saxagliptin 5mg + metformin 2000mg	9.2 (0.8)	6.3	2.9 (−3.1, −2.8)
		Saxagliptin 5mg + acarbose 300mg	9.2 (0.8)	6.6	2.6 (−2.8, −2.5)
		Saxagliptin 5mg + Gliclazide 120mg	9.2 (0.7)	6.4	2.8 (−2.9, −2.6)
Dou et al^25^	2018	Saxagliptin 5 mg + Metformin	9.4 (1.1)	6.4	3
		Metformin + placebo	9.5 (1)	6.7	2.8
		Saxagliptin 5 mg + placebo	9.4 (1)	7.3	2.1
Haak et al^26^	2012	Linagliptin 2.5 + metformin 1000mg	8.7 (1)	7.4 (0.1)	1.3
		Linagliptin 2.5 + metformin 2000mg	8.7 (1)	7.1 (0.1)	1.6
		Metformin 1000mg	8.7 (0.9)	8 (0.1)	0.7
		Metformin 2000mg	8.5 (0.9)	7.6 (0.1)	0.9
		Linaglipin 5mg	8.7 (1)	8.2 (0.1)	0.5
		Placebo	8.7 (1)	8.8 (0.1)	−0.1
Hadjadj et al.^27^	2016	Empagliflozin 12.5 mg b.i.d. + Metformin 1,000 mg b.i.d.	8.66 (1,14)	6.58	2.08
		Empagliflozin 12.5 mg b.i.d. + Metformin 500 mg b.i.d.	8.84 (1.31)	6.91	1.93
		Empagliflozin 5 mg b.i.d. + Metformin 1,000 mg b.i.d.	8.65 (1.23)	6.58	2.07
		Empagliflozin 5 mg b.i.d. + Metformin 500 mg b.i.d	8.68 (1.26)	6.7	1.98
		Metformin 1,000 mg b.i.d. Monotherapy	8.58 (1.13)	6.83	1.75
		Metformin 500 mg b.i.d. Monotherapy	8.69 (1.04)	7.51	1.18
		Empagliflozin 25 mg q.d. Monotherapy	8.86 (1.29)	7.5	1.36
		Empagliflozin 10 mg q.d. Monotherapy	8.62 (1.24)	7.27	1.35
Jadzinsky et al^28^	2009	Saxagliptin 5mg + metformin IR 500mg	9.4 (1.2)	6.9	2.5
		Saxagliptin 10mg + metformin IR 500mg	9.5 (1.2)	7	2.5
		Saxagliptin 10mg + placebo	9.6 (1.3)	7.9	1.7
		Metformin IR 500mg + placebo	9.4 (1.3)	9.5	−0.1
Ji et al^29^	2016	Sitagliptin 50 mg plus metformin 500 mg bd	8.7 (1.1)	7 (1.2)	1.7
		Sitagliptin 50 mg plus metformin 850 mg bd	8.5 (1)	6.8 (1)	1.7
		Metformin 1000mg	8.7 (1.1)	7.6 (1.5)	1.1
		Metformin 1700 mg	8.7 (1)	7.3 (1.1)	1.4
		Sitagliptin 100 mg	9 (1.1)	8.1 (1.8)	0.9
		Placebo	8.6 (0.9)	6.7 (1.3)	1.9
Ji et al^30^	2017	Alogliptin 25mg + metformin 1000mg	8.39 (0.81)	6.86	1.53
		Metformin 1000mg	8.4 (0.77)	7.36	1.04
		Alogliptin 25mg	8.48 (0.71)	7.62	0.86
		Placebo	8.21 (0.77)	8.02	0.19
Ji et al^31^	2015	Linagliptin 5mg + metformin 1000mg	8 (1)	7.01	0.99 (0.05)
		Metformin 2000mg	8 (0.8)	7.2	0.8 (0.04)
Jianfang et al^32^	2023	Pioglitazone 15mg + metformin 1000mg	8.13 (0.71)	6.48 (1.11)	1.65
		Metformin 1000mg	8.25 (1.02)	6.53 (0.93)	1.72
Lewin et al		Empagliflozin 25 mg/Linagliptin 5 mg	7.99 (0.95)	6.91	1.08
		Empagliflozin 10 mg/Linagliptin 5 mg	8.04 (0.96)	6.8	1.24
		Linagliptin 5mg	8.05 (0.89)	7.38	0.67
		Empagliflozin 25 mg Monotherapy	7.99 (0.97)	7.04	0.95
		Empagliflozin 10 mg Monotherapy	8.05 (1.03)	7.22	0.83
Mu et al ^34^	2017	Linagliptin 2.5/metformin 500mg bd	8.7 (0.9)	6.55	2.15
		Linagliptin 2.5/metformin 1000mg	8.7 (1)	6.41	2.29
		Metformin 500mg	8.7 (1)	7.06	1.64
		Metformin 1000mg BD	8.6 (1.1)	6.53	2.07
		Linagliptin 5mg	8.7 (0.9)	7.41	1.29
Pan et al ^35^	2021	Jinlinda granules (1 sachet) + metformin 1500mg	8.09 (0.8)	6.58 (0.61)	1.51
		Jinlinda granules (1 sachet)	7.83 (0.8)	7.15 (0.61)	0.68
		Metformin 1500mg + placebo	7.94 (0.63)	6.61 (0.57)	1.33
		Placebo only	7.88 (0.78)	7.43 (0.79)	0.45
Perez et al ^36^	2009	Pioglitazone 15 mg / metformin 850 mg taken twice daily	8.89 (0.99)	7.06	1.83
		Metformin 850 mg monotherapy taken twice daily	8.65 (1.01)	7.66	0.99
		Pioglitazone 15 mg monotherapy taken twice daily	8.69 (0.96)	7.73	0.96
Rosenstock et al ^37^	2016	Canagliflozin 100 mg + Metformin Group	8.8 (1.1)	7	1.8
		Canagliflozin 300 mg + Metformin Group	8.9 (1.2)	7	1.9
		Metformin Monotherapy	8.8 (1.2)	7.4	1.4
		Canagliflozin 100 mg Monotherapy	8.8 (1.2)	7.4	1.4
		Canagliflozin 300 mg Monotherapy	8.8 (1.2)	7.3	1.5
Rosenstock et al ^38^	2010	Colesevalam 3.75g/daily + metformin 1700mg	7.8 (1)	6.6	1.2
		Metformin 1700mg/placebo	7.5 (0.9)	6.7	0.8
Rosenstock et al ^39^	2010	Alogliptin 12.5 mg + Pioglitazone 30 mg	8.85 (1.039)	7.29	1.56
		Alogliptin 25 mg + Pioglitazone 30 mg	8.8 (0.962)	7.09	1.71
		Pioglitazone 30 mg	8.76 (1.005)	7.61	1.15
		Alogliptin 25 mg	8.8 (0.988)	7.84	0.96
Ross et al ^40^	2015	Linagliptin 5mg + metformin 2000mg	9.79 (1.19)	6.98	2.81 (0.12)
		Linagliptin 5mg	9.88 (1.1)	7.86	2.02 (0.13)
Tao et al ^41^	2018	Saxagliptin 5mg + metformin 2000mg	7.4 (0.3)	6.1 (0.4)	1.3
		Metformin 2000mg	7.3 (0.2)	6.3 (0.3)	1
		Saxagliptin 5mg	7.4 (0.3)	6.3 (0.2)	1.1
Wang et al ^42^	2011	Metformin 1500mg + repaglinide 3mg daily	10.91 (1.45)	6.4	4.51 (1.64)
		Repaglinide 3mg daily	10.73 (1.5)	6.68	4.05 (1.59)
Yoo et al ^43^	2021	Vidagliptin 100mg + metformin up to 2000mg	6.7 (0.4)	5.8	0.9
		Metformin 2000mg + Placebo	8 (0.4)	7.02	0.98

### 3.1 Risk of bias results

Risk of bias was assessed independently by two reviewers using Cochrane RoB 2 (2021) for individually randomised parallel‐group trials (*n* = 24 RCTs; 13,614 patients), focusing on HbA1c endpoints. Disagreements were resolved by consensus.

## 4 DISCUSSION

Our findings in newly or recently diagnosed patients with T2D provide supportive evidence that initial combination pharmacotherapy is superior to monotherapy (combined with lifestyle measures) for optimising HbA1c control. Across 20 randomised controlled trials comprising 37 arms, dual therapy achieved HbA1c ≤ 7.5% (58 mmol/mol) in 86% of arms, compared to 82% with monotherapy (OR 1.33, 95% CI 1.20–1.47, *p* = 0.002), demonstrating rapid and consistent attainment of targets that reduce therapeutic inertia. These findings are based on arm‐level meta‐analyses in which each arm entered a given comparison only once, including in multi‐arm trials, thereby preserving the independence of effect estimates within each contrast.

These benefits were observed across trial durations of 12–52 weeks and baseline HbA1c levels ranging from 6.5% to 11.0%, including patients with significant pre‐treatment hyperglycaemia, for whom monotherapy often proves less effective.

Dual therapy with metformin plus any additional agent was significantly more effective than metformin monotherapy (≤7.5% (58 mmol/mol): OR 1.39, *p* = 0.002), emphasising the limitations of metformin as a sole initial treatment, especially at higher baseline HbA1c values. Notably, combinations involving metformin and SGLT2‐i demonstrated the greatest efficacy, achieving superiority at all thresholds (≤7.5% (58 mmol/mol): 88% vs. 81%, OR 1.55, *p* ≤ 0.001; ≤ 7.0% (53 mmol/mol): OR 1.47, *p* ≤ 0.001; ≤ 6.5% (48 mmol/mol): OR 1.29, *p* = 0.030). SGLT2‐i inclusive dual therapy outperformed all monotherapy regimens (≤7.5% (58 mmol/mol): OR 1.43, *p* ≤ 0.001), while SGLT2‐i monotherapy itself surpassed metformin alone across all thresholds (≤7.5% (58 mmol/mol): 89% vs. 81%, OR 1.73, *p* ≤ 0.001). Importantly, dual therapy with SGLT2‐is showed no additional glycaemic benefit over SGLT‐2i monotherapy (≤7.5% (58 mmol/mol): OR 0.87, *p* = 0.480), positioning SGLT‐2is as highly potent standalone initial treatments.

In interpreting these findings, it is important to note that we treated SGLT2‐is as a uniform class. Prior large meta‐analyses have demonstrated modest differences in HbA1c‐lowering efficacy among individual SGLT2‐i agents, but the direction of effect is broadly consistent across the class.^11^, ^12^, ^13^ Within our dataset, the number of arms for each SGLT2‐i was too small to support robust agent‐level meta‐analyses across all three HbA1c thresholds. Heterogeneity within SGLT2‐i‐containing comparisons was low to moderate and the effect sizes were remarkably consistent, which supports a class‐level interpretation. Nevertheless, our results should be understood as reflecting the average performance of the SGLT2‐i class rather than any individual agent. The choice of a specific agent in clinical practice should continue to consider the broader evidence for cardiovascular and renal outcomes.

The magnitude of HbA1c reduction achieved with SGLT2‐i containing regimens in our review is consistent with the modest glycaemic potency reported in previous large meta‐analyses, in which SGLT2‐i typically lowers HbA1c by approximately 0.5 to 0.9 percentage points from baseline when added to background therapy.^11^, ^12^, ^13^ In our pooled analyses, the superiority of SGLT2‐i based strategies over metformin monotherapy is primarily reflected in a higher probability of attaining target thresholds rather than in very large absolute decrements in HbA1c. For example, SGLT2‐i monotherapy achieved HbA1c 7.5% in 88%–89% of patients, compared with around 81% with metformin monotherapy and metformin plus an SGLT2‐i achieved 88% at this threshold, versus 81% with metformin alone. These differences are clinically important because they shift a substantial proportion of patients across treatment targets, even though the underlying mean HbA1c reductions remain in the moderate range typical of oral glucose‐lowering agents.

When interpreted alongside published estimates for metformin, DPP‐4i, and sulfonylureas, these findings support the view that SGLT2‐i are moderate‐potency glucose‐lowering agents whose appeal derives from a favourable balance of glycaemic effects, weight and blood pressure benefits, plus proven cardiovascular and renal protection.

These findings align with and expand upon prior systematic reviews and meta‐analyses reporting glycaemic and weight reduction‐related advantages of early combination therapy.^12^, ^14^, ^15^, ^16^ Unlike earlier analyses, this study provides a detailed quantification of SGLT2‐i strategies across multiple clinically relevant HbA1c thresholds, incorporates data through to December 2025 and remains robust after excluding rosiglitazone‐containing trials (with no qualitative changes). This contemporary evidence supports recent guidelines advocating dual therapy at the time of T2D diagnosis, particularly for patients with baseline HbA1c ≥ 8.0% (64 mmol/mol), in whom monotherapy failure exceeds 60%.

Our findings also require careful clinical interpretation in individuals presenting with very high baseline HbA1c and features suggestive of relative insulin deficiency. The randomised trials included in this review generally enrolled adults with newly diagnosed or early T2D and excluded patients with extreme hyperglycaemia, overt catabolic symptoms or suspected type 1 diabetes. In routine practice, patients who present with marked hyperglycaemia, osmotic symptoms, weight loss or ketosis risk are often better served by initial insulin‐based regimens to restore metabolic stability, with metformin and SGLT‐2is introduced or optimised subsequently. In such cases, the potential risk of ketoacidosis associated with SGLT‐2is in susceptible phenotypes should be carefully considered. Our recommendation that SGLT‐2is, alone or in combination with metformin, can be considered as initial therapy is therefore intended for patients with newly diagnosed or early T2D without evidence of significant insulin deficiency or acute metabolic decompensation.

T2D constitutes a growing global health challenge associated with microvascular, macrovascular and renal complications.^17^ Early attainment of glycaemic targets confers long‐term benefits (the legacy effect of metabolic memory). Utilising data from the Royal College of General Practitioners Research and Surveillance Centre, Whyte et al. demonstrated that newly diagnosed T2D patients with baseline HbA1c ≥ 75 mmol/mol (9.0%), who achieved sustained HbA1c ≤ 58 mmol/mol (7.5%) experienced a 25% reduction in major adverse cardiovascular events (HR 0.75, 95% CI 0.60–0.94, *p* = 0.014).^18^ Our findings suggest that dual therapy facilitates prompt target achievement across diverse severities, directly addressing therapeutic inertia that often delays intensification.

Real‐world comparative effectiveness data further support SGLT2 inhibitors as preferred adjuncts to metformin.^19^ Carroll et al. reported that second‐line SGLT2 inhibitors, combined with metformin, outperform sulfonylureas or DPP‐4i over two years in reducing HbA1c, BMI, systolic blood pressure, heart failure hospitalisations (compared to DPP‐4 inhibitors) and decline in eGFR by 40% or more (compared to sulfonylureas).^20^ Significantly, these glycaemic benefits persisted despite nearly 40% of patients presenting with renal impairment at diagnosis, which lessens the glucose‐lowering capacity of SGLT‐2is.

While glucagon‐like peptide‐1 (GLP‐1) and glucose‐dependent insulinotropic polypeptide (GIP) receptor agonists, with or without metformin, constitute potent initial pharmacotherapeutic options, their costs are frequently prohibitive within the constraints of the NHS and global health budgets. Our findings endorse the utilisation of metformin in conjunction with SGLT‐2is or SGLT‐2i monotherapy, as effective and cost‐efficient alternatives.

### 4.1 Strengths and limitations

This PROSPERO‐registered review includes comprehensive searches through to December 2025, meticulous duplicate data extraction, seven prespecified contrast analyses across monotherapy classes and combinations, multiple HbA1c thresholds, sensitivity analyses excluding rosiglitazone and low‐to‐moderate heterogeneity (*I* ^2^ = 25–50%). Limitations include a focus solely on glycaemic outcomes (excluding cardiovascular endpoints), heterogeneity in patient populations despite consistent findings, variable trial durations (12–52 weeks) and the exclusion of insulin regimens. Nonetheless, this represents the most current and thorough synthesis of evidence supporting early dual therapy in T2D management.

The exclusion of injectable GLP‐1 receptor agonists is acknowledged as a limitation, particularly given their established efficacy and their role in contemporary treatment algorithms.

A further limitation is heterogeneity in metformin dosing and titration strategies across the included trials. Metformin monotherapy arms spanned a range of total daily doses, and metformin‐containing dual regimens used several fixed and titrated dosing schedules. As dose bands were not reported consistently enough to define homogeneous subgroups, we treated metformin monotherapy as a single comparator category and did not undertake dose‐stratified analyses. It is therefore possible that higher‐dose metformin regimens could narrow, although they are unlikely to abolish, the advantages we observed for dual therapy and SGLT‐2i based strategies. Future studies with individual‐patient data would be better placed to model metformin dose–response relationships more precisely.

In conclusion, this systematic review and meta‐analysis provide definitive evidence that initial dual oral therapy achieves better glycaemic control compared to monotherapy across several HbA1c thresholds in treatment‐naive and early T2D patients. Dual therapy achieved HbA1c ≤ 7.5% (58 mmol/mol) in 86% of interventions, compared with 82% with monotherapy (OR 1.33, *p* = 0.002), with consistent benefits at ≤7.0% and notable trends at ≤6.5%. The combination of metformin and SGLT‐2is demonstrated the greatest efficacy (≤7.5%: OR 1.55, *p* ≤ 0.001), while SGLT‐2i monotherapy exceeded metformin alone (OR 1.73, *p* ≤ 0.001). Importantly, SGLT‐2i dual therapy showed no added advantage over SGLT‐2i monotherapy, establishing SGLT2 inhibitors as potent initial agents.

These findings may have important clinical implications. Early dual therapy enables rapid attainment of glycaemic targets, reduces therapeutic inertia associated with stepwise escalation and harnesses the legacy effect for ongoing cardiovascular protection. Given the rising global prevalence of T2D, prioritising regimens that achieve ≥85% target success at diagnosis is a strategic use of resources. SGLT‐2is may emerge as the first‐line option surpassing metformin monotherapy, even in the context of prevalent renal impairment at diagnosis.

Current guidelines, including those from the American Diabetes Association,^6^ recognise dual therapy as an appropriate initial strategy in many individuals with newly diagnosed T2D, particularly to shorten the time to glycaemic target attainment. SGLT‐2is are prioritised in specific populations (e.g., heart failure, chronic kidney disease), but other oral and injectable agents are also considered effective options.

These findings may inform future guideline updates endorsing metformin combined with SGLT‐2is or SGLT‐2is as monotherapy, as preferred initial treatment, SGLT‐2is or SGLT‐2is as monotherapy, for appropriately selected patients without features of insulin deficiency or acute metabolic decompensation, especially for patients with baseline HbA1c 8.0% (64 mmol/mol) or less.

This synthesis of evidence supports a shift from traditional metformin‐centric approaches toward outcome‐driven pharmacotherapy, moving management of T2D from reactive to proactive, with early combination therapy serving as the new standard of care.

## AUTHOR CONTRIBUTIONS

AH, MBW and HLE conceived the study and designed the protocol in consultation with MK and SJ. MK and SS performed data processing and data analysis in consultation with AH and MBW. AH and MK wrote the first draft of the manuscript. All authors reviewed the manuscript during its development, approved the final version and agree to be accountable for all aspects of the work.

## FUNDING INFORMATION

This research did not receive any specific grant from funding agencies in the public, commercial, or not‐for‐profit sectors.

## CONFLICT OF INTEREST STATEMENT

None of the authors has any conflicts of interest to declare.

## ETHICS STATEMENT

Ethical approval was not required as the analysis involved the use of pooled data from already published primary research papers.

## GUARANTOR STATEMENT

AH is the guarantor of this work and, as such, has full access to all the data in the study and takes responsibility for the integrity of the data and the accuracy of the data analysis.

## Supporting information


**Supplementary Figure 1.** Summary of Risk of Bias Judgements. Bar chart summarising risk of bias across 24 randomised controlled trials (*n* = 13,614 patients) using Cochrane RoB 2 (2021). Proportions reflect judgments for HbA1c endpoints across five domains: (1) bias arising from the randomisation process; (2) bias due to deviations from intended interventions; (3) bias due to missing outcome data; (4) bias in measurement of the outcome; (5) bias in selection of the reported result. Green = low risk; yellow = some concerns; red = high risk. Universal low risk was observed for outcome measurement (Domain 4; 100%) due to central laboratory HbA1c analysis. Primary concerns were deviations from interventions (Domain 2; 42% some concerns, driven by open‐label designs) and missing data (Domain 3; 33% combined concerns/high risk). Overall judgements: 21% low risk (*n* = 5), 33% some concerns (*n* = 8), 46% high risk (*n* = 11). Sensitivity analyses excluding high‐risk studies were consistent with primary analyses.

## Data Availability

The data that support the findings of this study are available on request from the corresponding author. The data are not publicly available due to ethical restrictions.
