# Supplementary material for: A systematic review and meta‐analysis of the relative benefit of dual versus single oral hypoglycaemic therapy following diagnosis with type 2 diabetes (T2D)
Source: Diabet Med. 2026 May 21;43(7):e70337. doi: 10.1111/dme.70337 (PMC13257891; doi:10.1111/dme.70337)
Supplement: Supplementary file 1 — Supplementary Figure 1. Summary of Risk of Bias Judgements. Bar chart summarising risk of bias across 24 randomised controlled trials (n = 13,614 patients) using Cochrane RoB 2 (2021). Proportions reflect judgments for HbA1c endpoints across five domains: (1) bias arising from the randomisation process; (2) bias due to deviations from intended interventions; (3) bias due to missing outcome data; (4) bias in measurement of the outcome; (5) bias in selection of the reported result. Green = low risk; yellow = some concerns; red = high risk. Universal low risk was observed for outcome measurement (Domain 4; 100%) due to central laboratory HbA1c analysis. Primary concerns were deviations from interventions (Domain 2; 42% some concerns, driven by open‐label designs) and missing data (Domain 3; 33% combined concerns/high risk). Overall judgements: 21% low risk (n = 5), 33% some concerns (n = 8), 46% high risk (n = 11). Sensitivity analyses excluding high‐risk studies were consistent with primary analyses. [file DME-43-e70337-s001.doc]

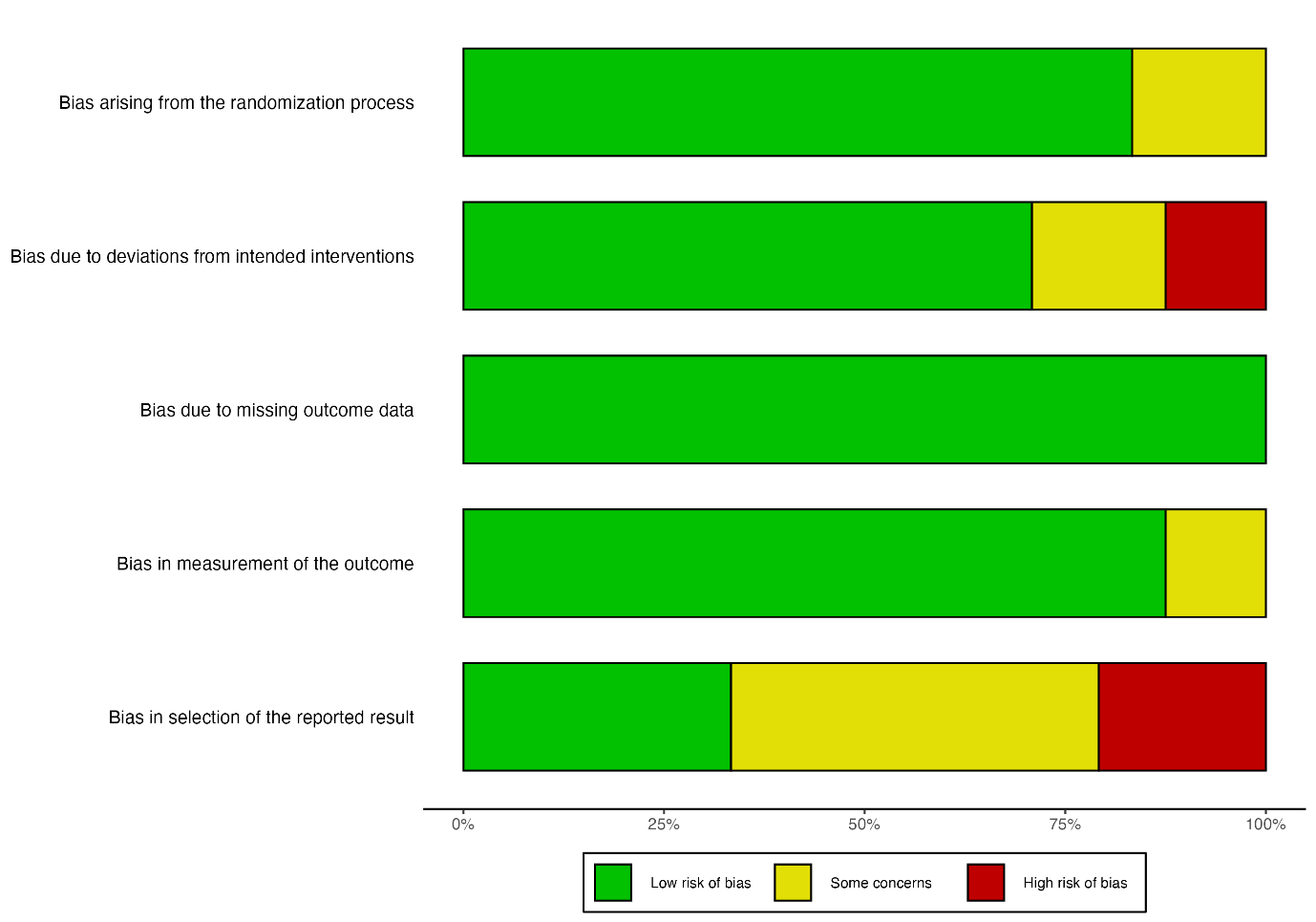


Supplementary Figure 1. Summary of Risk of Bias Judgements. Bar chart summarising risk of bias across 24 randomised controlled trials (n=13,614 patients) using Cochrane RoB 2 (2021). Proportions reflect judgments for HbA1c endpoints across five domains: (1) bias arising from the randomisation process; (2) bias due to deviations from intended interventions; (3) bias due to missing outcome data; (4) bias in measurement of the outcome; (5) bias in selection of the reported result. Green = low risk; yellow = some concerns; red = high risk. Universal low risk was observed for outcome measurement (Domain 4; 100%) due to central laboratory HbA1c analysis. Primary concerns were deviations from interventions (Domain 2; 42% some concerns, driven by open-label designs) and missing data (Domain 3; 33% combined concerns/high risk).​ Overall judgements: 21% low risk (n=5), 33% some concerns (n=8), 46% high risk (n=11). Sensitivity analyses excluding high-risk studies were consistent with primary analyses.
